# Further delineation of EBF3-related syndromic neurodevelopmental disorder in twelve Chinese patients

**DOI:** 10.3389/fped.2023.1091532

**Published:** 2023-03-03

**Authors:** Jitao Zhu, Wenhui Li, Sha Yu, Wei Lu, Qiong Xu, Sujuan Wang, Yanyan Qian, Qiufang Guo, Suzhen Xu, Yao Wang, Ping Zhang, Xuemei Zhao, Qi Ni, Renchao Liu, Xu Li, Bingbing Wu, Shuizhen Zhou, Huijun Wang

**Affiliations:** ^1^ Center for Molecular Medicine, Pediatrics Research Institute, Children’s Hospital of Fudan University, National Children’s Medical Center, Shanghai, China; ^2^ Neurology Department, Children’s Hospital of Fudan University, National Children’s Medical Center, Shanghai, China; ^3^ Department of Endocrinology and Inherited Metabolic Diseases, Children’s Hospital of Fudan University, National Children’s Medical Center, Shanghai, China; ^4^ Department of Child Health Care, Children’s Hospital of Fudan University, National Children’s Medical Center, Shanghai, China; ^5^ Department of Rehabilitation, Children’s Hospital of Fudan University, National Children’s Medical Center, Shanghai, China

**Keywords:** neurodevelopmental disorders (NDDs), exome sequencing, *EBF3* gene, novel variants, copy number variations (CNVs)

## Abstract

Neurodevelopmental disorders (NDDs) have heterogeneity in both clinical characteristics and genetic factors. *EBF3* is a recently discovered gene associated with a syndromic form of NDDs characterized by hypotonia, ataxia and facial features. In this study, we report twelve unrelated individuals with *EBF3* variants using next-generation sequencing. Five missense variants (four novel variants and one known variant) and seven copy number variations (CNVs) of *EBF3* gene were identified. All of these patients exhibited developmental delay/intellectual disability. Ataxia was observed in 33% (6/9) of the patients, and abnormal muscle tone was observed in 55% (6/11) of the patients. Aberrant MRI reports were noted in 64% (7/11) of the patients. Four novel missense variants were all located in the DNA-binding domain. The pathogenicity of these variants was validated by *in vitro* experiments. We found that the subcellular protein localization of the R152C and F211L mutants was changed, and the distribution pattern of the R163G mutant was changed from even to granular. Luciferase assay results showed that the four EBF3 mutants' transcriptional activities were all significantly decreased (*p* < 0.01). Our study further expanded the gene mutation spectrum of EBF3-related NDD.

## 1. Introduction

Neurodevelopmental disorders (NDDs) account for a significant proportion of congenital disorders, which impose an enormous financial burden on families and society. Researches have suggested that hundreds of genes are involved in the pathogenesis of NDDs, but the underlying mechanisms remain unclear (1). Affected individuals present with various neurological symptoms, including developmental delay (DD), intellectual disability (ID), autism spectrum disorder (ASD), epilepsy and other minor symptoms such as decreased pain sensitivity or hyperactivity (2, 3). In addition, patients may also present with phenotypes in other systems, such as congenital heart defects, skeletal or muscular abnormalities, metabolic disorders, gastrointestinal problems, distinctive facial features or strabismus, etc. Despite the high heterogeneity of pathogenic genes and the diverse clinical features of NDDs, advances in next-generation sequencing technology have facilitated the diagnosis of such diseases (4, 5).

*EBF3* is one of the causative genes that lead to syndromic NDDs. The earliest reports of EBF3-related NDD dated back to 2017, in which a total of 21 cases were summarized and analyzed (6–8). In their description, multiple systems were involved, including central nervous system (CNS), genitourinary system, skeletal system, etc. Major phenotypes included DD/ID, ataxia, hypotonia, structural CNS malformations, genitourinary abnormalities, subtle facial features, and strabismus. Other less common phenotypes included dysarthria, constipation, decreased pain sensitivity during development, and behavioral deficits such as attention deficit and ASD or ASD-like symptoms. Since then, more than 30 additional cases of EBF3-related NDDs have been reported (9–16). A recent meta-analysis integrated previously published 42 cases with detailed patient information and their 41 new cases, and quantified the risk and severity of patient phenotypes based on these 83 patients (17). The author concluded that *EBF3* missense variants affecting the zinc knuckle motif (ZNK, a domain located in the DNA-binding domain of the EBF3 protein) carried a higher risk subtype of the disease.

This study identified five single nucleotide variants (SNVs), including four novel missense variants and one previously reported missense variant, and seven copy number variants (CNVs). We validated the pathogenicity of the four novel variants by functional experiments and summarized the correlation between the genotypes and phenotypes of our patients.

## 2. Materials and methods

### 2.1. Clinical exome sequencing (CES) and exome sequencing (ES)

This study was approved by the ethics committees of Children's Hospital, Fudan University (2016-235 and 2022-331). Pre-test counseling was provided, and patients' informed consent was obtained from at least one parent. Between February 2, 2016, and March 3, 2022, over forty thousand patients were included in the analysis, and approximately three thousand patients were found to have NDDs. Genomic DNA was extracted from peripheral blood collected from the patients and their parents using the QIAamp DNA Blood Mini Kit under the manufacturer's instructions. The library was constructed and sequenced as 150-bp paired-end runs on the Illumina X Ten platform. The Agilent ClearSeq inherited disease panel kit was used in CES, and the Agilent SureSelect XT Human all exon 50 Mb kit was used in ES (18). Sequencing was conducted following the protocols described in our published work (19).

### 2.2. Read mapping, variant calling and interpretation

Low quality reads were discarded from raw data to generate clean reads. Clean reads were then aligned to a modified human reference genome (UCSC hg19). Sorting, merging, and removing duplicates were then performed, and single nucleotide variants were called using the Genome Analysis Toolkit Best Practices Pipeline. Allele frequencies for variants were annotated according to gnomAD, the ExAC, 1,000_genome and our in-house database. The remaining variants were then computationally compared with mutations reported in the Human Gene Mutation Database (HGMD). Variants reported in the HGMD were retained if they had a minor allele frequency of less than 5% according to either the 1,000_genome or the ExAC database. For genomic changes not reported in the HGMD, synonymous variants, intronic variants that were more than 15 bp from exon boundaries, and common variants (minor allele frequency >1%) were discarded. SIFT, PolyPhen-2 and MutationTaster were used to predict the pathogenicity of the variants. The variant assessment followed the American College of Medical Genetics standards and guidelines for data interpretation (20).

For CNV analysis, within the BAM file from the same sequencing batch, exon coverage depth was calculated, and quality control was performed. CANOES was used to calculate scores and provide candidate CNVs (21) which were then annotated and filtered at the genetic and regional levels. Refseq, Online Mendelian Inheritance in Man (OMIM), HGMD and Swiss Prot were used as references for gene-level annotation. Gene deletions/duplications of high frequency (occurrence in the internal samples >10%) were filtered out. After automatic matching with the standard Human Phenotype Ontology, CNVs that affected gene function and had relevant phenotypes consistent with previous literature were annotated. The Database of Genomic Variants, DECIPHER, the Database of Chromosomal Imbalance and Phenotype in Humans using Ensemble Resources, and previous literature reporting pathogenic key genes were combined and used as references for region-level annotation (Dong et al., 2020). With reference to the key genes in pathogenic CNVs reported or summarized in previous literature, known genes that were likely to cause disease *via* the form of CNV were annotated. In addition, CNVs larger than 1 Mb were also annotated. The CNVs were manually evaluated by taking into account the inheritance patterns of the key genes, previous literature, ClinGen curation, the matching degree of patients' phenotype, and the frequency and size of the CNVs.

### 2.3. Validation by Sanger sequencing

Sanger sequencing was performed to confirm the genetic mutations. Primer 5.0 was used to design the primers (WES-EBF3-487-F: 5′-TGACTCCTACCGCTTCATTGTC-3′, WES-EBF3-487-R: 5′-TTGTTGCTGCTGCGGTTTT-3′, WES-EBF3-188-F: 5′-GGCCGCTGTCTCTTTCCTC-3′, WES-EBF3-188-R: 5′-GAGAGAGGGTGTGATCGTGTG-3′, WES-EBF3-422-F: 5′-GGAGGCAGGGGAGGGAGAA-3′, WES-EBF3-422-R: 5′-CCTCGGGCCGATTACATTTCA-3′, WES-EBF3-631-F: 5′-TGAGTCTTTTGTCTGATAACCCTAATAAA-3′, WES-EBF3-631-R: 5′-CCTGAACAGTTGCAAATCAGAGA-3′, WES-EBF3-454-F: 5′-TAACCCCAGCCTCTGCTTGT-3′, WES-EBF3-454-R: 5′-ATTTCAATCGATGCCCTTCC-3′). The following analysis was performed using MutationSurveyor (SoftGenetics, State College, PA, United States).

### 2.4. Average face construction

We selected one frontal 2D photograph with a neutral expression for each patient. The photographs were anonymized to ensure confidentiality. We then ran a pre-trained deep learning model from Face++ (https://www.faceplusplus.com) to place 106 landmarks on each photograph. Finally, we constructed an average face from the three sets of photographs and landmarks using standard morphing procedures of OpenCV in Python3.7 as recommended (https://learnopencv.com/average-face-opencv-c-python-tutorial/).

### 2.5. Prediction on the effect of the variants on EBF3-DNA interactions

The sequences of the DNA-binding domain (DBD) (from 26-240aa) in EBF1 and EBF3 were 94.9% identical. Therefore, we used the structural model of EBF1 bound to DNA (PDB: 3MLP) as the structure template (22). DBePISA webserver (http://pdbe.org/pisa/) was used to predict the hydrogen bonds between wild-type/mutant EBF3 and the DNA. PyMOL was used to visualize the protein-DNA complex and the internal interactions within the complex.

### 2.6. Plasmid construction

To further analyze the pathogenicity of the *EBF3* variants, subcellular localization analysis and dual luciferase reporter assays were conducted. The *EBF3* coding sequence was inserted into pEGFP-N1-3xFlag to construct the pEGFP-N1-EBF3-3xFlag expression vector using the Hieff Clone Plus One Step Cloning Kit (Cat No. 10911). The EBF3 mutant plasmids were constructed by site-directed mutagenesis (KOD-Plus-Mutagenesis Kit). Since EBF3 mediates the transcription of the p21 (23), we also constructed the pGL3-p21-Luc reporter plasmid to determine whether *EBF3* mutations impair the transactivation capacity of its target gene p21. The following primer sequences were used for mutagenesis: EBF3-R63Q-F: 5′-AGAAATCCAATTTCTTCCACTTCGTGC-3′, EBF3-R63Q-R: 5′-GGAGGTTGGAAGGCGGCTGCTTCTCGA-3′, EBF3-R152C-F: 5′-TGTGTGCTGCTGACCCACGAGAT-3′, EBF3-R152C-R: 5′-GCACATCTCCGGGTTCTTGTCCT-3′, EBF3-R163G-F: 5′-TCTTGTCACAGCACCCGCTGCACATGATCTC-3′, EBF3-R163G-R: 5′-GAGATCATGTGCAGCGGGTGCTGTGACAAGA-3′, EBF3-F211L-F: 5′-CTCCAGGTTGTTGTATCGACAACA-3′, EBF3-F211L-R: 5′-TCTCCGCATATCTCGAGGGTT-3′.

### 2.7. Subcellular localization analysis

Hela cells were cultured in DMEM containing 10% fetal bovine serum with 1% penicillin and streptomycin. For subcellular localization analysis of wild-type and mutant EBF3 protein, cells were transfected with the expression constructs of wild-type or mutant EBF3 tagged with GFP. Forty-eight hours after transfection, cells were fixed, incubated with DAPI for 5 min, embedded in a mounting medium, and then analyzed by confocal imaging system using Leica TCS SP8. The Fiji software was used to profile the pixels of the restricted area to provide a more visual description of the relative positions of the DAPI and GFP-tagged EBF3 proteins (24). Pearson's correlation coefficients were calculated and then used in the bar chart to illustrate the extent of change in the overlap between DAPI and GFP-tagged EBF3 protein (25).

### 2.8. Luciferase reporter assay

Cells were transfected with different expression plasmids of EBF3, together with pGL3-p21-Luc and pREN at a ratio of 25:25:1 (pGL3-p21-Luc encodes firefly luciferase, and pREN encodes Renilla luciferase). Cell lysates were used to detect dual luciferase activity 48 h after transfection. The activity of Renilla luciferase was used to normalize the data, and the basal promoter activity for transfection with pEGFP-N1 empty vector was used as control.

## 3. Results

### 3.1. Clinical characteristics of the twelve patients

**Figure 1:**
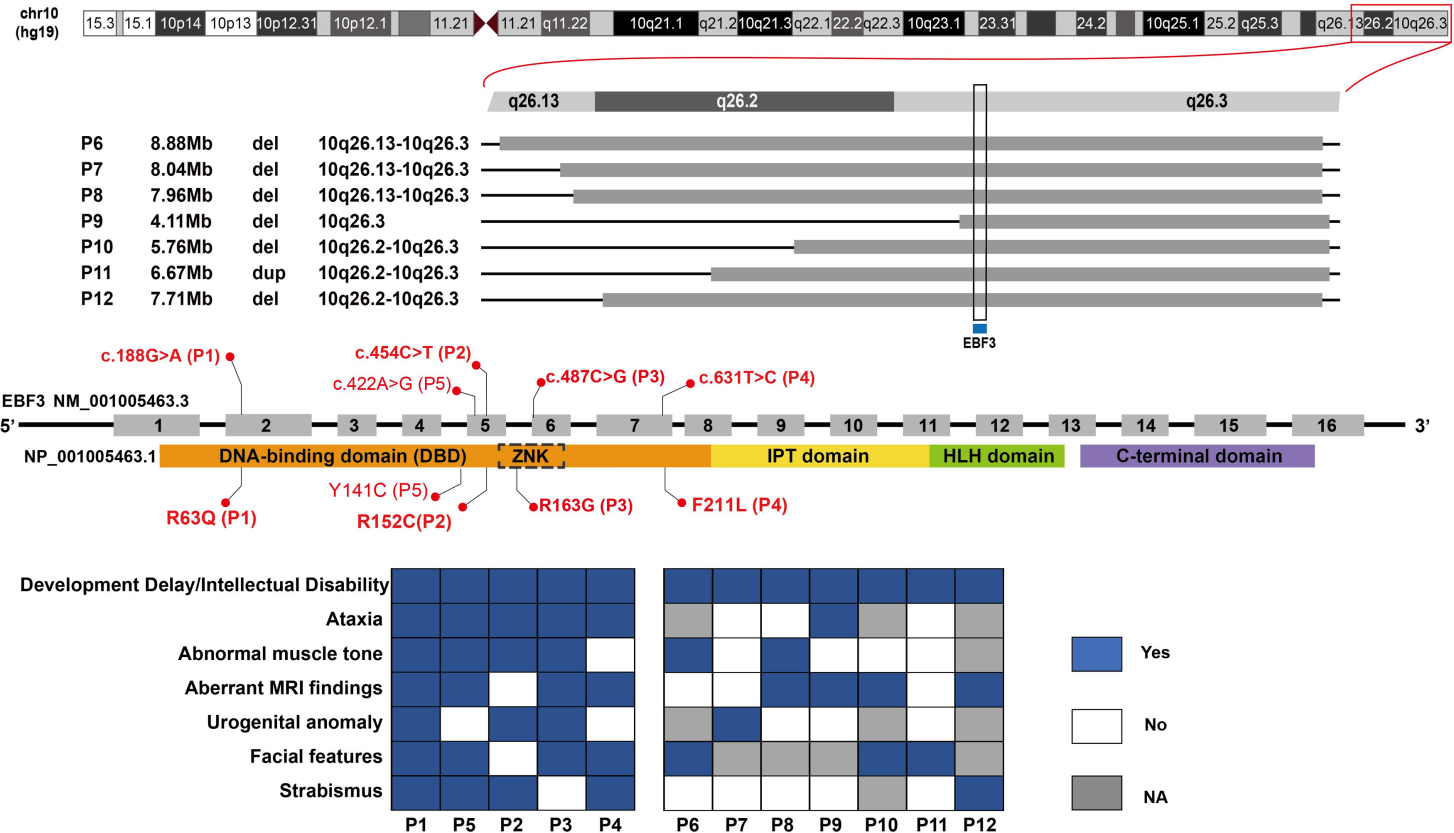
*EBF3* gene variations and clinical phenotypes of patients in our study. Five single nucleotide variants are shown along the *EBF3* gene structure in the middle; the base changes are shown above the *EBF3* exon-intron structure and the corresponding amino acid changes are shown below the protein domains. The bold red ones are novel, and the normal red one is reported. The chromosome structure and the seven copy number variants are shown above the gene structure. The genotype-phenotype correlation is shown below the gene structure. A blue square box indicates that the patient has the phenotype, a white square box indicates that the patient does not have the phenotype and a gray square box indicates that the patient's phenotype is not available.

Twelve patients were detected with *EBF3* gene variations (Figure 1), including seven males and five females, with ages at genetic tests ranging from 9 months to 6 years old and the median age of 2 years and 10 months (Table 1). Facial characteristics of typical triangular-shaped long face, ocular hypertelorism and flat nasal bridge were noted in 87.5% (7/8) of patients. A corresponding average facial image constructed from the collected frontal facial photographs is shown in Supplementary Figure S1. P3, in particular, had a myopathic appearance with limited facial expression and seldom smiled. Strabismus was observed in 33% (4/12) of the patients.

**Table 1:** Clinical information of the twelve patients with *EBF3* variants in this study.

Patient	P1	P2	P3	P4	P5	P6	P7	P8	P9	P10	P11	P12	Summary
Sex	M	F	M	M	F	F	F	F	M	M	M	M	7M, 5F
Age (at genetic test)	9 months	4 years	1 year 9 months	1 year	4 years 8 months	6 years	3 years	2 years 6 months	2 years 1 months	2 years 11 months	2 years 10 months	2 years 9 months	2 years 10 months (md)
Variant	c.188G > A p.R63Q	c.454C > T p.R152C	c.487C > G p.R163G	c.631T > C p.F211L	c.422A > G p.Y141C	10q26.13–10q26.3 8.88Mb del	10q26.13–10q26.3 8.04Mb del	10q26.13–10q26.3 7.96Mb del	10q26.3 4.11Mb del	10q26.2–10q26.3 5.76Mb del	10q26.2–10q26.3 6.67Mb dup	10q26.2–10q26.3 7.71Mb del	5 SNVs, 7 CNVs
Inheritance	*De novo*	NA	*De novo*	*De novo*	*De novo*	*De novo*	*De novo*	NA	NA	NA	NA	NA	6 *De novo*
Protein domain	DBD	DBD	ZNK	DBD	DBD	–	–	–	–	–	–	–	5 SNVs in DBD (1 in ZNK)
gnomAD/ExAC/1,000_genome	0/0/0	0/0/0	0/0/0	0/0/0	0/0/0	–	–	–	–	–	–	–	0/0/0 in 5 SNVs
SIFT/PP2/MT	D/D/D	D/D/D	D/D/D	D/P/D	D/P/D	–	–	–	–	–	–	–	D/P in 5 SNVs
CADD	30	31	34	28.2	30	–	–	–	–	–	–	–	30.64 (avg) in 5 SNVs
REVEL	0.592	0.607	0.537	0.688	0.651	–	–	–	–	–	–	–	0.615 (avg) in 5 SNVs
Class and evidence	LP PS2 + PM2_P + PP3_M	VUS PM2_P + PP3_M	LP PS2 + PM5 + PM2_P + PP3_M	LP PS2 + PM2_P + PP3_M	P PS2 + PS4 + PS3?+PM2_P + PP3_M	*P* ≥ 0.99	*P* ≥ 0.99	*P* ≥ 0.99	*P* ≥ 0.99	*P* ≥ 0.99	VUS 0.75	*P* ≥ 0.99	7 P, 3 LP, 2 VUS
Facial features	+	–	+	+	+	+	NA	NA	NA	+	+	NA	7/8
Strabismus	+	+	–	+	+	–	–	–	–	–	–	NA	4/11
Head circumference	47 (9 months) (>1SD)	NA	47 (3 years 5 months) (<1SD)	38.3 (4 months) (<3SD)	NA	46.5(6 years) (<1SD)	NA	NA	NA	NA	NA	NA	4/4 abnormal
Height	103 (5 years 11 months) (<2SD)	NA	NA	65 (4 months) (normal)	NA	108 (6 years) (<1SD)	84 (3 years) (<2SD)	NA	NA	NA	NA	NA	3/4 abnormal
Neuromuscular Findings													
Developmental delay	+	+	+	+	+	+	+	+	+	+	+	+	12/12
Developmental quotient	45 (6 months)	NA	NA	62 (16 months)	59 (5 years)	NA	NA	63 (30 months)	47 (19 months)	50 (35 months)	49 (34 months)	47 (33 months)	49.5 (md)
Speech delay	+	–	+	+	–	NA	+	–	+	NA	+	NA	6/9
Ataxia	+	+	+	+	+	NA	–	–	+	NA	–	NA	6/9
Abnormal muscle tone	+ (9 months),−(5 years)	+ (4 years),−(6 years)	+ (6 months),−(7 years)	–	+ (4 years 8 months), - (7 years 5 months)	+ (6 years)	–	+ (2 years 6 months) - (6 years 2 months)	–	–	–	NA	6/11
Aberrant MRI reports	+	–	+	+	+	–	–	NA	+	+	–	+	7/11
Attention deficit	+	+	+	+	+	NA	–	–	–	NA	–	NA	5/9
Hyperactivity	+	–	–	+	–	NA	–	–	–	NA	–	NA	2/9
Stereotypy	–	–	+	–	–	NA	–	–	–	NA	–	NA	1/9
Poor eye contact	–	–	+	–	–	NA	–	–	+	NA	+	NA	3/9
Limited social participation	–	-	+	–	–	NA	–	–	+	NA	+	NA	3/9
Other findings													
Urogenital abnormalities	+	+	+	–	–	NA	+	–	–	NA	–	NA	4/9
Constipation	+	+	+	–	–	NA	–	–	–	NA	–	NA	3/9
Decreased pain sensitivity	+	+	–	+	–	NA	–	–	–	NA	–	NA	3/9
Teeth and skeletal system	Flat feet;	–	Flat feet; pectus excavatum	–	Dysplasia of teeth and gum; flat feet	–	–	Mild scoliosis	–	–	–	–	4/12

M, male; F, female; y, year; m, month; +, yes; −, no; NA, not available; md, median; avg, average.

DBD, DNA-binding domain; ZNK, zinc knuckle motif.

SIFT/PP2/MT, the assessment on SNVs by softwares SIFT, PolyPhen-2 and MutationTaster; D, damaging; P, probably damaing;.

P, pathogenic; LP, likely pathogenic; VUS, variant of unknown significance.

All patients failed to meet the motor and language developmental milestones for their age group. Gesell Developmental Schedules were used to evaluate the developmental quotient (DQ) of the patients. The DQ was calculated as follows: DQ = [developmental age (DA)] divided by [chronologic age (CA)] × 100. The median DQ for the eight patients with quantitative assessments was 49.5. Ataxia was observed in 6/9 (67%) of the patients who mainly presented with wobbly and unsteady gait and a tendency to fall. Abnormal muscle tone was noticed in 6/11 (55%) patients. Increased muscle tone in the upper limbs was observed in P3; intermittent increased muscle tone was observed in P5; generalized increased muscle tone was observed in P6. Decreased tendon reflex and positive pathological reflex were reported in P1. Hypotonia was reported in P1, P2 and P8. Aberrant magnetic resonance imaging (MRI) reports were noted in 7/11 (64%), indicating a possible mild structural abnormality in brain development. Widening of the extracerebral space and/or fullness in the lateral ventricle were reported in P3, P4, P5, P10 (Supplementary Figure S2A,B). Widened sulci were reported in P1. Hypoplasia of the splenium of the corpus callosum was reported in P9 at one and a half years old (Supplementary Figure S2C,D). Multiple T2-FLAIR high signals in bilateral parietal white matter with fullness in bilateral ventricles were reported in P12 at the age of 2 years and 9 months (Supplementary Figure S2E,F). Attention deficit was reported in 5/9 (56%) of the patients who presented mainly with a short attention span. Hyperactivity was observed in 2/9 (22%) patients who showed constant activity and aggressiveness. Stereotypy was observed in P3 (1/9, 11%), while poor eye contact and limited social participation were observed in 3/9 (33%). P3 and P11 were clinically suspected of having ASD, and P11 received an Autism Diagnostic Observation Schedule test with a score of 15 for social emotions and 1 for stereotypy.

Other clinical features were observed in seven patients. Urogenital deficits were reported in 4/9 (44%) of the patients. Among them, male patients (P1 and P3) were found with cryptorchidism, while female patients were found with congenital ureteral dilatation in P2 and neurogenic bladder and vesicoureteral reflux in P7. Both constipation and decreased pain sensitivity were noticed in P1 and P2. P3 has constipation and P4 has decreased pain sensitivity. However, these two symptoms appeared in infancy and waned with age in these four patients. Other less common manifestations included dental and skeletal dysmorphism. Moderate dental hypoplasia was noticed in P5, as she had brittle front teeth and presented exposed tooth roots without the rest part of the teeth. Flat feet were observed in 3 patients, P1, P2 and P5. Pectus excavatum was noticed in P3. Mild scoliosis was noted in P7.

In the follow-up, the intelligence quotient for P1 at age five was 77 and for P7 at age six was 61. The mild to moderate degree of their intellectual disability may be due to the fact that these two patients started to receive rehabilitation at a very young age and continued in training. Speech delay was found in 67% (6/9) of the patients, and slurred speech (dysarthria) was particularly noted in three patients (P1, P3 and P6). In terms of abnormal muscle tone, the physician carried out a physical examination on P1, P2 and P3 at the follow-up visit, and the results showed that their muscle tone had returned to normal. Telephone follow-up with the parents indicated that P5 and P8 had also returned to normal muscle tone with no abnormalities.

### 3.2. Variations of *EBF3* gene identified in twelve patients with syndromic NDD

Next generation sequencing identified twelve patients with *EBF3* gene variations, including five SNVs and seven CNVs (Table 1 and Figure 1). Of the five identified SNVs, Y141C found in P5 was recorded as a pathogenic variant in ClinVar and HGMD, and the other four identified in P1-P4 were novel variants (c.188G > A, p.R63Q; c.454C > T, p.R152C; c.487C > G, p.R163G; c.631T > C, p.F211L). These four missense variants were not reported in gnomAD, 1,000_genome databases, our in-house database, or any published literature. The prediction results of PolyPhen-2, SIFT and MutationTaster were all damaging or probably damaging. Three variants had a CADD score above 30, while c.487C > G had a score of 28.3. Two variants had a REVEL score above 0.6, while c.188G > A had a score of 0.592 and c.487C > G had a score of 0.537. Parental testing confirmed that c.188G > A, c.487C > G and c.631T > C were *de novo* variants, while parental samples of the P2 with c.454C > T were not available. Three variants (c.188G > A, c.487C > G and c.631T > C) were classified as likely pathogenic and c.454C > T was classified as a variant of unknown significance (VUS).

The seven CNVs were identified in P6-P12, including six deletions and one duplication, ranging in size from 4.11 Mb to 8.88 Mb (Supplementary Figure S3, S4). These seven CNVs were mainly located between 10q26.13 and 10q26.3 on the chromosome 10. They all covered the *EBF3* gene, which was of autosomal dominant inheritance and associated with developmental delay in OMIM (Supplementary Table S1). All these seven CNVs were classified as pathogenic or likely pathogenic (Table 1).

### 3.3. Phenotype and genotype analysis of the patients with *EBF3* gene variants

In our study, five patients carried missense variants, all of which were located in the DBD (R163G fell within the ZNK motif). The individual difference in phenotypes among these patients with SNVs was subtle (Figure 1, P1–P5). They mainly presented DD/ID, ataxia, abnormal muscle tone, aberrant MRI findings, urogenital abnormalities, similar facial features including typical triangular-shaped long face, ocular hypertelorism, flat nasal bridge, and strabismus. In patients with CNVs (Figure 1, P6–P12), the phenotype predominantly involved DD/ID, with other phenotypes scattered across various systems and the distribution patterns varying from patient to patient.

### 3.4. In silico analysis of the four novel *EBF3* missense variants

**Figure 2:**
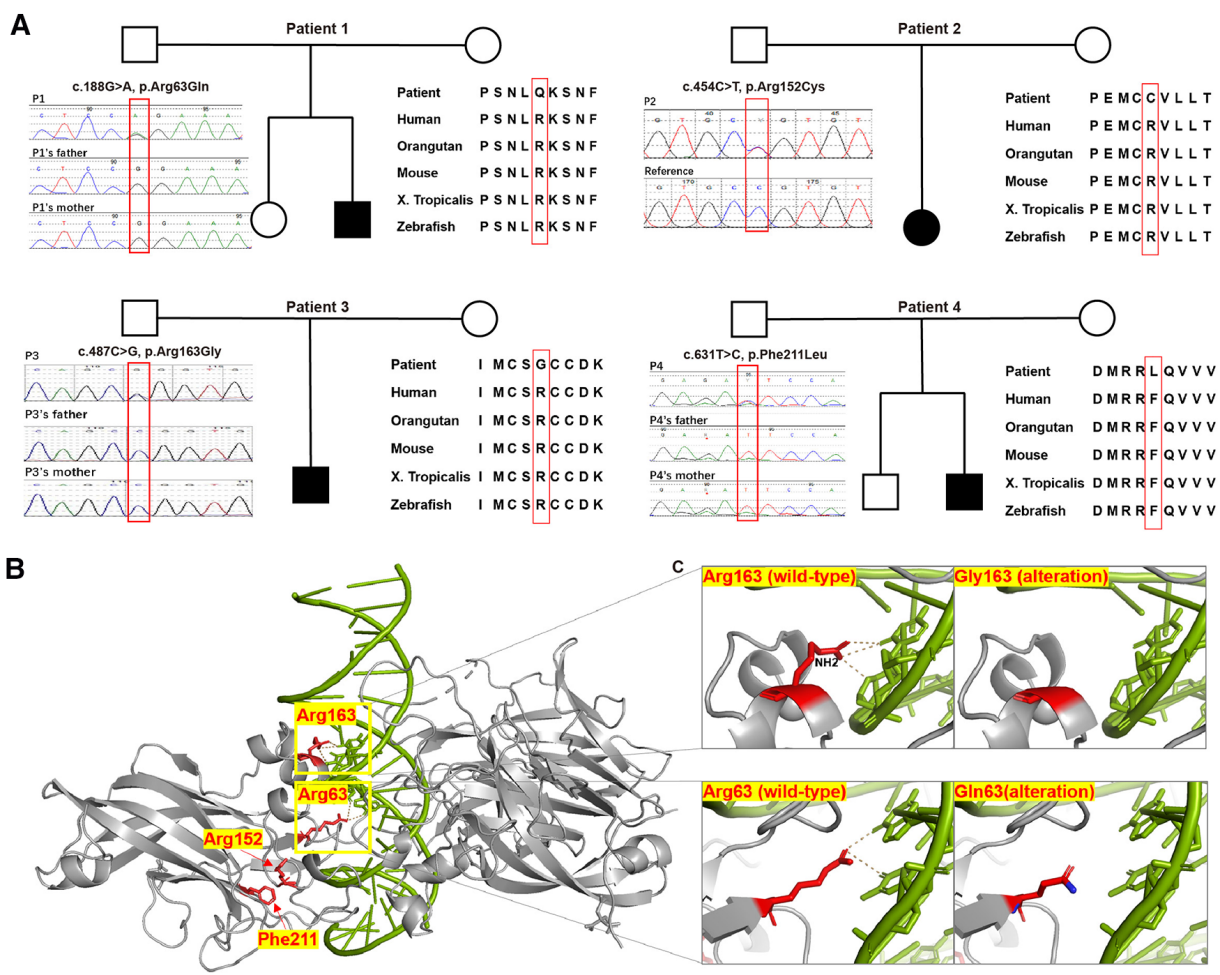
Pedigree chart, evolutionary conservation, Sanger sequencing results, protein structure model, and close-ups of novel variants detected in our patients. (**A**) Pedigree charts of the patients with novel *EBF3* single nucleotide variants and the corresponding evolutionary conservation and Sanger sequencing results in our study. (**B**) Model of the DBD of an EBF3 dimer (gray ribbon; affected residues are shown as red sticks) bound to DNA (shown as a green duplex; DNA residues involved in hydrogen bonding with the affected protein residues are shown as sticks). (**C**) Close-up view of the effect of Arg163Gly and Arg63Gln alterations on hydrogen bond formation.

The four novel missense variants are all located in highly conserved sites across species (Figure 2A). In the 3MLP protein structure model, we can see both Arg63 and Arg163 are close to the DNA helix (Figure 2B). The Arg163 forms the hydrogen bond with DNA *via* the amino group -NH2 (Figure 2C, the top panel), and the R163G alteration was predicted to directly affect the EBF3-DNA binding. Replacement of Arg with Gly resulted in the loss of the NH2 group and could severely interfere with hydrogen bond formation, which would likely damage the stabilization of the EBF3-DNA complex. The R63Q alteration was also predicted to have an impact on EBF3-DNA binding. The substitution of Arg with Gln resulted in a longer distance between the residue and DNA, thus weakening the hydrogen bond between Arg63 and DNA (Figure 2C, the bottom panel). The R152C and F211L alterations were not involved in the participation of hydrogen bond formation, although they are also close to the DNA helix.

### 3.5. In vitro functional experiments of the four novel *EBF3* missense variants

**Figure 3:**
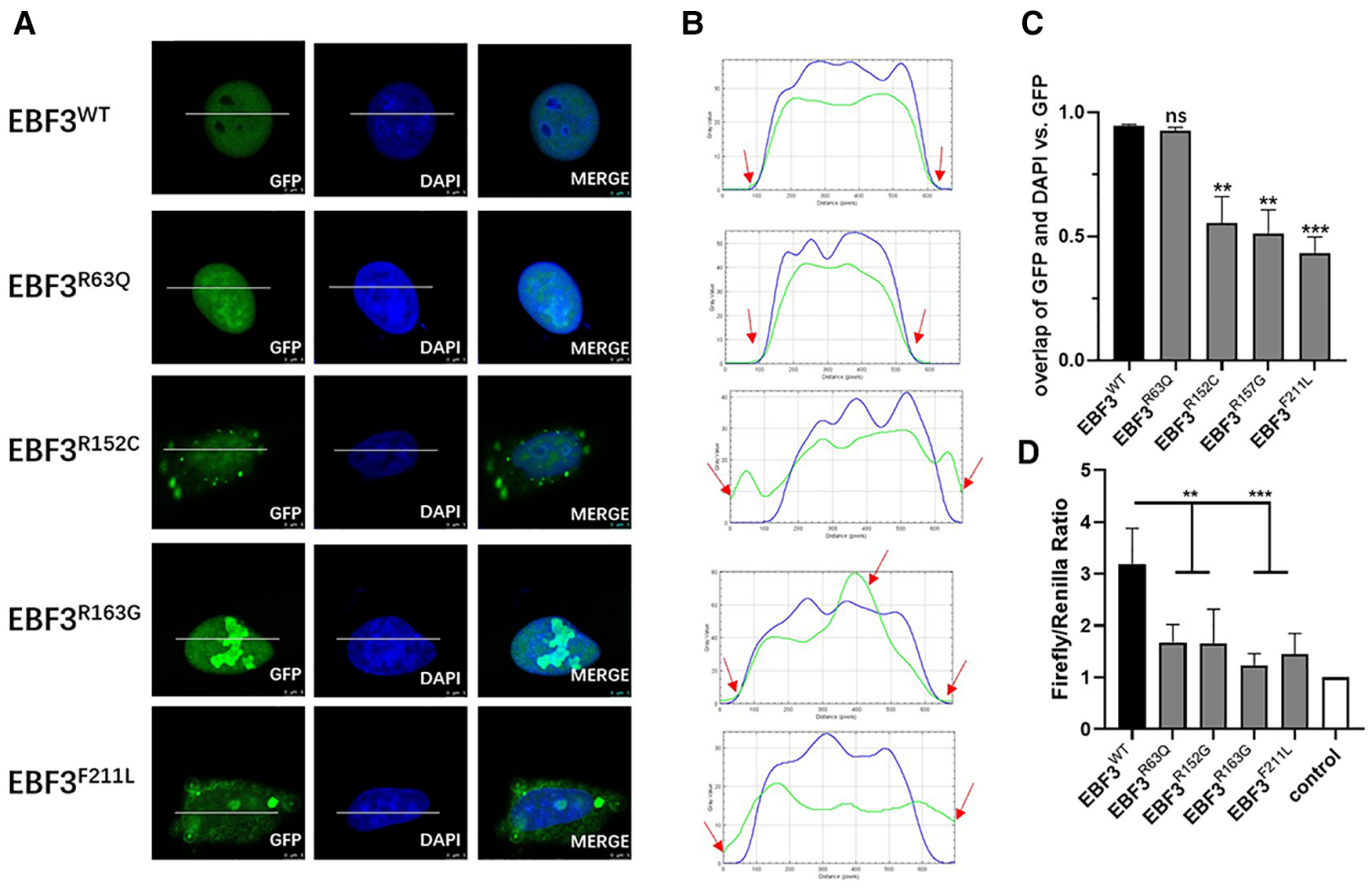
In vitro functional analysis of four novel missense variants at the cellular level. (**A**) Localization of GFP-EBF3-WT and GFP-EBF3-Mut (R63Q, R152C, R163G and F211L) in Hela cells. (**B**) Intensity profiles of GFP-tagged EBF3 wild-type and mutants and the DNA-binding dye, DAPI. The horizontal coordinate represents the pixels on the white lines drawn in A. The vertical coordinate represents the fluorescence intensity of the pixel. The green curve represents the intensity profile of GFP-EBF3, and the blue curve represents the intensity profile of DAPI. For each condition a representative image and the corresponding intensity profile plot are shown. (**C**) The Pearson’s correlation coefficient is calculated for colocalization analysis. Three representative single cell images from three individual experiments are analyzed using coloc2 (a plugin in the Fiji software) and the results are shown as mean ± SD. **p < 0.01, ***p < 0.001. (**D**) Dual luciferase assay results reflect the expression of the luciferase activated by p21 promoter bound with EBF3-R63Q, EBF3-R152G, EBF3-R163G or EBF3-F211L. Results are mean ± SD for four individual experiments which were performed in triplicate for each condition. ns: not significant, **p < 0.01, ***p < 0.001.

In Hela cells, compared with the overexpressed GFP-fusion wild-type protein, the subcellular location of the R63Q and R163G GFP-fusion mutant proteins were not changed. However, expression of the R152C and F211L mutant proteins was also observed in the cytoplasm. Interestingly, the expression density of the R152C, R163G, and F211L mutant proteins was not evenly distributed in the nucleus and in the cytoplasm, but showed a granular pattern, especially in the nucleus of cells expressing R163G mutant protein (Figure 3A).

Figure 3B further confirmed these observations by showing the intensity profiles of the GFP-fusion EBF3 wild-type and variants and the DNA binding dye DAPI. In the wild-type and R63Q groups, a similar intensity trend was observed for both the green and the blue curves. In the R152C and F211L groups, the green curve rose earlier and descended later than the blue curve, suggesting cytoplasmic expression in addition to nuclear expression. However, in the R163G group, there was an apparent peak in the green curve compared to a relatively flat blue curve, suggesting a high density of the mutant protein.

Coloc2 in the Fiji software was used to calculate the correlation coefficient of DAPI and GFP-fusion EBF3, and the results are shown in Figure 3C. The overall spatial relationship between R63Q and DNA remained insignificantly changed, while the colocalization between R152C, R163G, or F211L with the DNA was significantly reduced. Taken together, these results show that *EBF3* mutations lead to changes in subcellular localization.

A dual luciferase reporter assay was conducted to further evaluate whether EBF3 mutants would still regulate the transcription of p21. The luciferase activity, normalized by the ratio of firefly to Renilla, was considered as the transcriptional activity of p21 promoter to eliminate the system error introduced by transfection. The value of the basal promoter activity for transfection with empty vector, together with pREN and pGL3-p21-Luc was considered as 1 (control). As shown in Figure 3D, the luciferase activity in the EBF3-WT group was approximately 3- to 4-fold higher than that of the empty vector, while the luciferase activities in the four EBF3 mutant groups were all significantly reduced (**p < 0.01, ***p < 0.001).

In summary, R163G affected the EBF3 protein distribution pattern while R152C and F211L also affected the subcellular localization. All four novel missense variants significantly impaired the regulatory activity of EBF3 in the transcription of p21. Taken together, these results validated the pathogenicity of the four novel *EBF3* missense variants.

## 4. Discussion

Patients with EBF3-related NDD mainly manifest DD/ID, ataxia, hypotonia, often accompanied by subtle facial features, strabismus, genitourinary abnormalities and structural abnormalities (6–8). Our patients also manifested similar phenotypes. All of our patients presented with DD/ID. Ataxia and abnormal muscle tone were mainly absent in our patients with CNVs. Aberrant MRI findings were found in patients with missense variants as well as in patients with CNVs. Urogenital abnormalities, facial features and strabismus were absent in our patients with CNVs. Due to the small number of patients and the wide age range, the average facial image cannot represent all the characteristics of the patients in our study. However, it may reflect to some extent the facial features that we have summarized: typical triangular long face, ocular hypertelorism, flat nasal bridge and strabismus.

In the functional experiments, we observed altered subcellular localization and distribution pattern of the EBF3 mutant proteins, and also found that the transactivation activity of the EBF3 mutants towards the p21 promoter was significantly impaired. Previous research showed that the EBF3 mutant proteins affected by variants located in the DBD expressed both in the cytoplasm and the nucleus, and the distribution of the mutant proteins was generally even without apparent clusters (7). In our study, EBF3 R152C and F211L mutant proteins showed similar expression patterns, present in both the nucleus and the cytoplasm. EBF3 R163G mutant protein showed a new granular expression pattern, although it was still exclusively located in the nucleus. We speculate that the altered distribution pattern is involved in the pathogenic mechanisms of mutant proteins, although there has been no further report on the subcellular location and distribution of other mutants harboring mutations on the same amino acid position. We hypothesized that the severity of the phenotype would vary depending on the location of the point mutations in the structural domain of the proteins, as we found that patients with R63Q, Y141C and R163G had a more severe phenotype than those with other missense variants in our report. Based on our prediction, R63Q and R163G could impair the hydrogen bond formation between EBF3 and DNA. In addition, according to previous literature (7), Y141C could result in a structural change at the dimer interface, which could compromise the stability of EBF3 dimer and thus the binding between them.

Recently, we noticed that the variants of c.188G > A (p.R63Q), and c.454C > G (p. R152G) were submitted to the ClinVar database by other researchers and were classified as likely pathogenic, pathogenic, respectively. In our study, we reported the same variant of c.188G > A (p.R63Q), and c.454C > T (p.R152C), a different nucleotide and amino acid change in the same site. Our analysis and experimental results were consistent with their classification, adding to their credibility. In addition, c. 633C > A (p. F211L) and c.633C > G (p. F211L) were also submitted to the ClinVar database by other researchers and were classified as VUS. We reported c.631T > G (p.F211L), which had a different nucleotide change resulting in the same amino acid change. Our experimental results added to the evidence that supported the pathogenicity of the amino acid change from Phe to Leu at 211.

The mechanism underlying how *EBF3* variants cause developmental disorders remains to be elucidated. One study found that when wild-type EBF3 was co-expressed with EBF3 mutans of N66D, Y141C, H157_I159dup, R303* or Q305*, a significant 40%–50% reduction in the reporter activity was observed, compared to the reporter activity in cells expressing the same amount of wild-type EBF3 protein alone (7). This suggests a potential dominant negative effect of these EBF3 mutants. Patients with large 10qter deletions or whole gene deletions covering EBF3 shared main clinical characteristics with EBF3-related NDD, such as DD and/or ID, hypotonia, strabismus, and triangular face (10, 12, 26–30), which supported haploinsufficiency as one of the possible pathogenic mechanisms of the *EBF3* mutations. As there has been one reported case of duplication/triplication mosaicism of EBF3, combined with the duplication we reported in this study, we speculate that triplosensitivity may also help to illustrate how the *EBF3* gene mutations causes disease. However, there is no curation for the *EBF3* gene in the GlinGen database, and the triplosensitive genes recorded in the ClinGen Genome Dosage Map are limited (31). Recently, a research has managed to work out a reliable framework to evaluate genetic dosage sensitivity. The prediction for EBF3 is 1.0 for pHaplo, and 0.996 for pTriplo, suggesting that the *EBF3* gene is both haploinsufficient and triplosensitive (32).

We speculate that the phenotype of patients with *EBF3* whole gene deletion and those with missense mutations may be similar, whereas in our study the phenotypes differed between patients with SNVs and those with deletions, and the latter appeared to have a mild and sporadic phenotype. This difference in phenotypic severity may be due to the following reasons: the CNVs reported in our study varied in size and contained different genes, so the phenotypes of patients carrying CNVs may be more heterogeneous, involving different systems; although *EBF3* was the known OMIM gene associated with DD, there may still be some genes covered in the CNVs that need further evaluation; most patients with CNVs were very young, whereas some phenotypes (e.g., ataxia, attention deficit, etc.) may not become apparent untill the patients are older, and therefore long-term follow-up was in need; the follow-up of patients with CNVs was done by telephone, and therefore some physical examinations and tests may not be available, and thus leading to a bias in the phenotypes.

In conclusion, our study identified twelve Chinese patients of syndromic NDD with *EBF3* variants, and we provided functional evidence for the four novel missense variants. In addition, we reported for the first time the phenotype of dysarthria and scoliosis in the Chinese population. Our study has further expanded the molecular and phenotypic spectrum of EBF3-related NDD. We also recommend regular long-term follow-up of patients with EBF3-related NDD.

## Data Availability

Variants detected in this study have been submitted to ClinVar with accession numbers SCV002564425.1, SCV002564427.1, SCV002564424.1 and SCV002564426.1. The raw data are not publicly available due to ethical restrictions.
