# Supplementary material for: Further delineation of EBF3-related syndromic neurodevelopmental disorder in twelve Chinese patients
Source: Front Pediatr. 2023 Mar 3;11:1091532. doi: 10.3389/fped.2023.1091532 (PMC10020332; doi:10.3389/fped.2023.1091532)
Supplement: Supplementary file 1 [file Datasheet1.docx]

Supplementary Material

Further delineation of EBF3-related syndromic neurodevelopmental disorder in twelve Chinese patients

**Jitao Zhu^1†^, Wenhui Li^2†^**, Sha Yu^1^, Wei Lu^3^, Qiong Xu^4^, Sujuan Wang^5^, Yanyan Qian^1^, Qiufang Guo^1^, Suzhen Xu^1^, Yao Wang^1^, Ping Zhang^1^, Xuemei Zhao^1^, Qi Ni^1^, Renchao Liu^1^, Xu Li^1^, Bingbing Wu^1*^, Shuizhen Zhou^2*^, Huijun Wang^1*^

^1^Center for Molecular Medicine, Pediatrics Research Institute, Children's Hospital of Fudan University, National Children's Medical Center, Shanghai 201102, China

^2^Neurology Department, Children's Hospital of Fudan University, National Children's Medical Center, Shanghai 201102, China

^3^Department of Endocrinology and Inherited Metabolic Diseases, Children's Hospital of Fudan University, National Children's Medical Center, Shanghai 201102, China

^4^Department of Child Health Care, Children's Hospital of Fudan University, National Children's Medical Center, Shanghai 201102, China

^5^Department of Rehabilitation, Children’s Hospital of Fudan University, National Children's Medical Center, Shanghai 201102, China

**†These authors contributed equally to this work and shared the first authorship**

***Correspondence:**

Bingbing Wu

[081107271@fudan.edu.cn](mailto:)

Shuizhen Zhou

szzhou@shmu.edu.cn

Huijun Wang

[huijunwang@fudan.edu.cn](mailto:huijunwang@fudan.edu.cn)


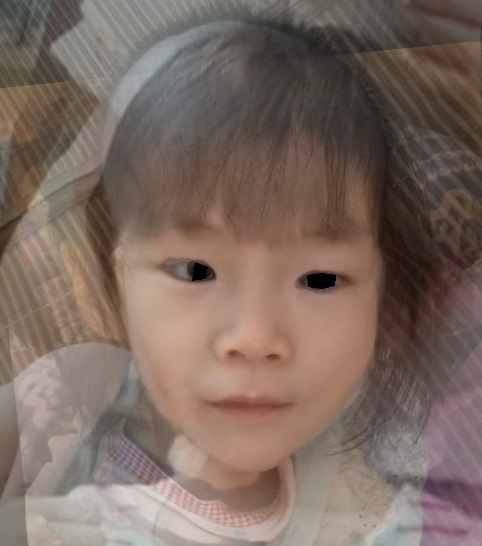


**Supplementary Figure S1.** The average facial image is based on the frontal facial photographs of our patients (P1, P5, P6), showing similar facial features, including a typical triangular-shaped long face, ocular hypertelorism, flat nasal bridge, and strabismus.


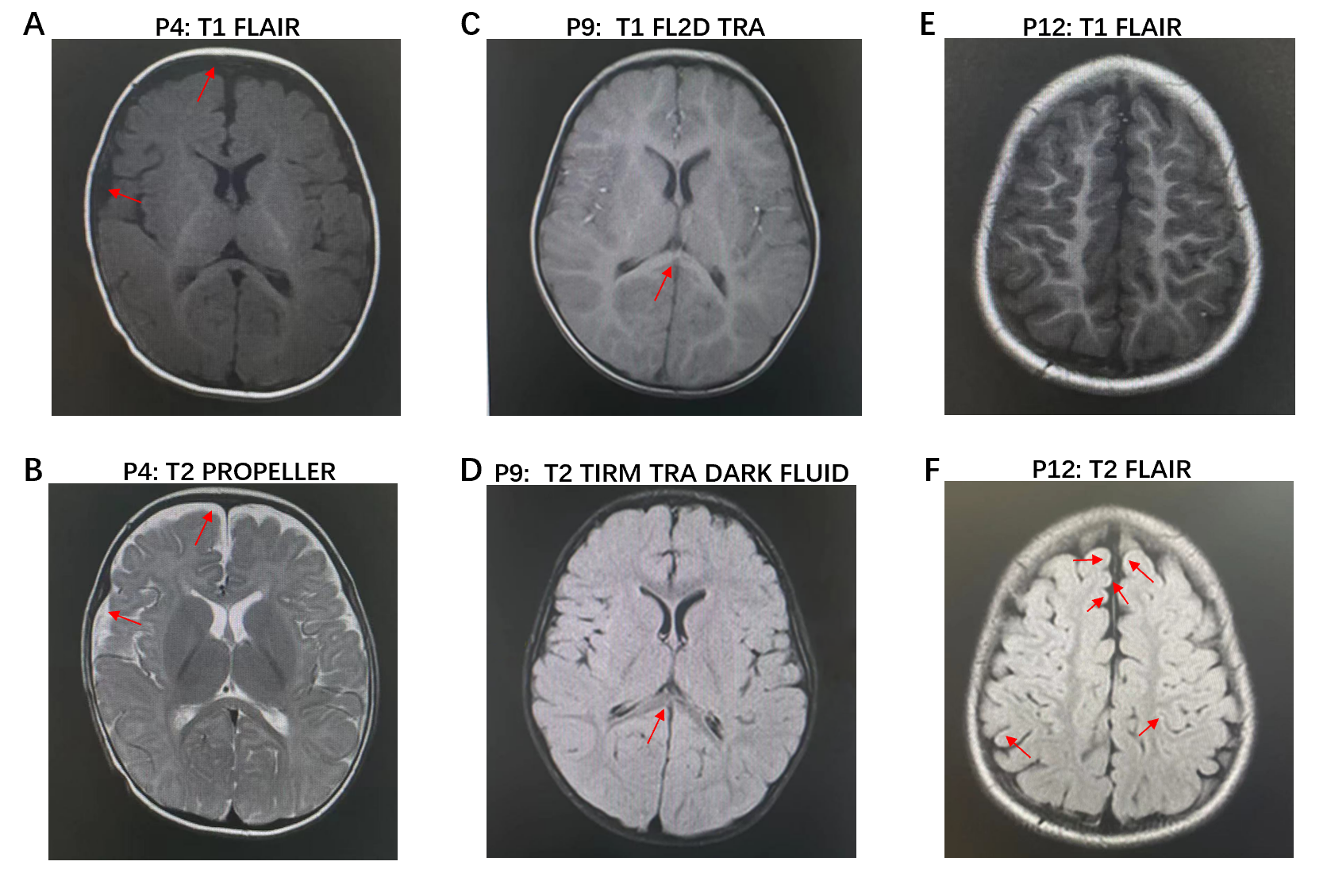


**Supplementary Figure S2** **Representative brain MRI images of P4, P9 and P12.** (A-B) Areas marked with red arrows suggested the widening of the extracerebral space in P4. (C-D) Areas marked with the red arrows indicated hypoplasia of the splenium of the corpus callosum in P9. (E-F) Areas marked with red arrows showed the high signal intensity in bilateral parietal white matter on T2-FLAIR images in P12.


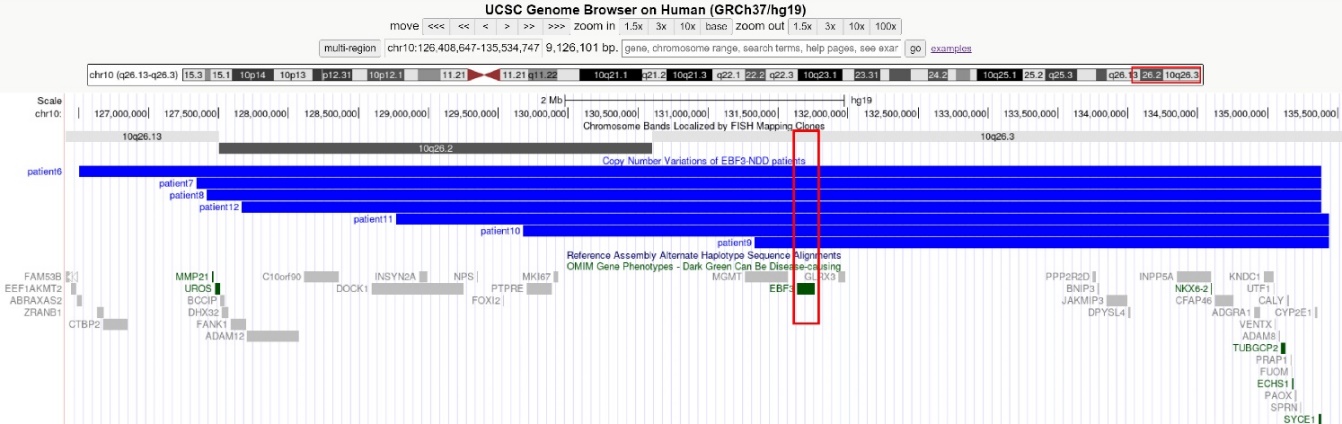


**Supplementary Figure S3.** The regions covered by CNVs detected in our patients are shown in the UCSC browser.


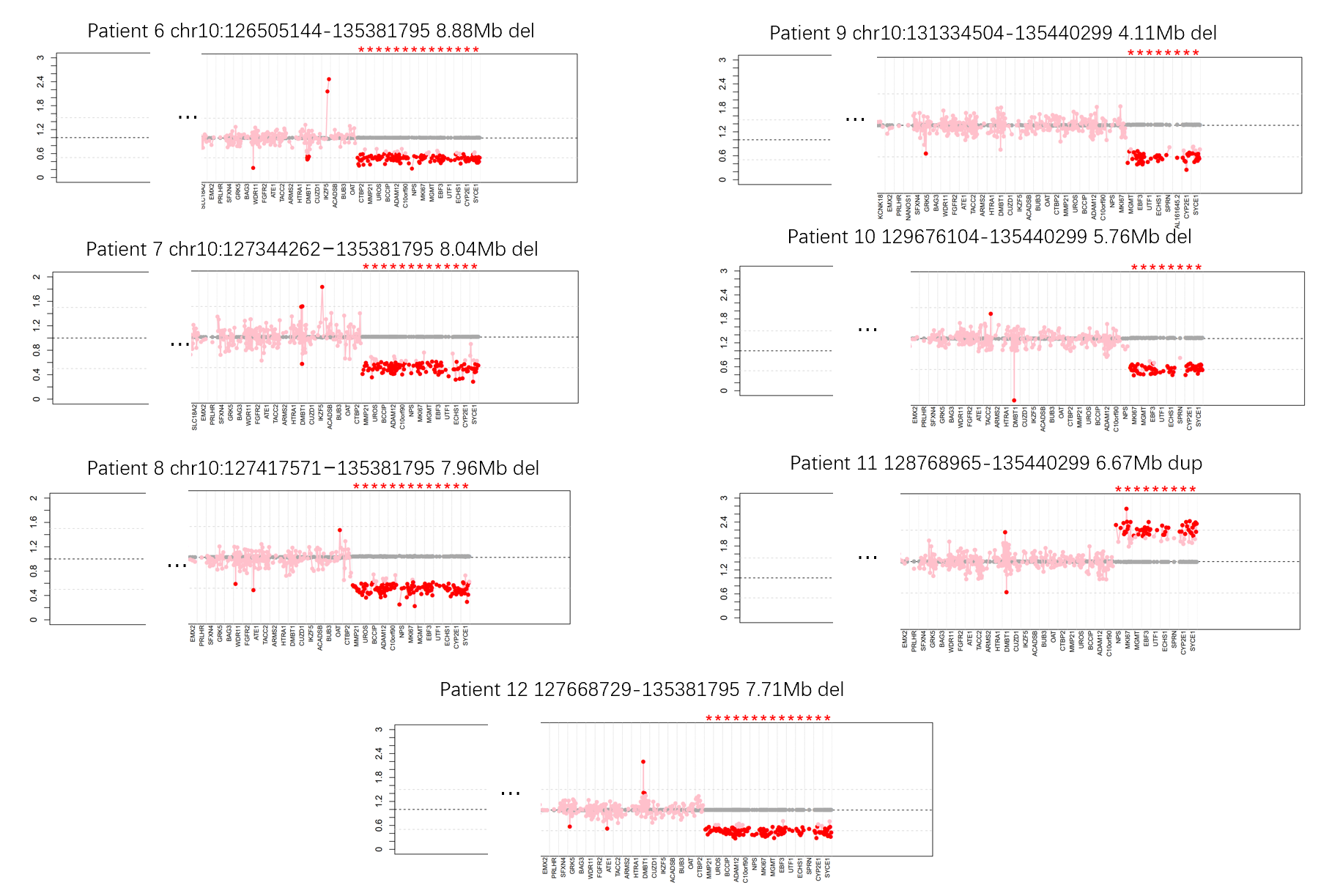


**Supplementary Figure S4**. The deletions/duplication regions and covered genes detected in our patients (digitally mapped from Bam files)

**Supplementary Table S1**. The genomic coordinates and genes covered in seven patients detected with CNVs.

| **Patient** | **Genomic coordinates (hg19)** | **Del/Dup** | **Gene number** | **OMIM gene number** | **pLI>0.9 & pHaplo>0.86;**  **pTriplo>0.94** | **Phenotype-matched disease gene** |
| --- | --- | --- | --- | --- | --- | --- |
| 6 | chr10:126505144-135381795 | del | 96 | 40 | ZRANB1, EBF3, INPP5A | EBF3 |
| 7 | chr10:127344262-135382916 | del | 88 | 37 | EBF3, INPP5A | EBF3 |
| 8 | chr10:127417571-135381795 | del | 84 | 37 | EBF3, INPP5A | EBF3 |
| 9 | chr10:131334504-135440299 | del | 58 | 24 | EBF3, INPP5A | EBF3 |
| 10 | chr10:129676104-135440299 | del | 63 | 26 | EBF3, INPP5A | EBF3 |
| 11 | chr10:128768965-135440299 | dup | 69 | 30 | EBF3 | EBF3 |
| 12 | chr10:127668729-135381795 | del | 74 | 33 | EBF3, INPP5A | EBF3 |

**Note:** del, deletion; dup, duplication.
